# Recent Progress in Artificial Neurons for Neuromodulation

**DOI:** 10.3390/jfb15080214

**Published:** 2024-07-30

**Authors:** Qinkai Jiang, Mengwei Liu

**Affiliations:** 1 College of Materials Science and Engineering, Sichuan University, Chengdu 610065, China; jiangqinkai@stu.scu.edu.cn; 2 School of Communication and Electronic Engineering, East China Normal University, Shanghai 200241, China

**Keywords:** artificial neurons, bio-integrated system, multi-modal sensing, implantable devices, neuromodulation

## Abstract

Driven by the rapid advancement and practical implementation of biomaterials, fabrication technologies, and artificial intelligence, artificial neuron devices and systems have emerged as a promising technology for interpreting and transmitting neurological signals. These systems are equipped with multi-modal bio-integrable sensing capabilities, and can facilitate the benefits of neurological monitoring and modulation through accurate physiological recognition. In this article, we provide an overview of recent progress in artificial neuron technology, with a particular focus on the high-tech applications made possible by innovations in material engineering, new designs and technologies, and potential application areas. As a rapidly expanding field, these advancements have a promising potential to revolutionize personalized healthcare, human enhancement, and a wide range of other applications, making artificial neuron devices the future of brain-machine interfaces.

## 1. Introduction

As one of the main organs in the human body, the brain plays a significant role in people’s daily activities. It consists of billions of neurons interconnected in a sophisticated neural network. Brain–machine interfaces (BMIs) have the ability to bridge the gap between humans and machines by interpreting and transmitting neurological information [1]. This is crucial in areas such as neuron rehabilitation, brain signal decoding, and body control [2,3,4]. Over the past few decades, people have been trying to understand the complex operation of the brain, which involves hundreds of millions of neurons working closely. These efforts have led to a number of exciting applications, yet not enough to acquire a full understanding of the brain’s functional wiring diagram, which hinders the development of all related fields.

Currently, the artificial neuron has recently gained significant attention in the field of BMIs, as it allows for the sensing and interpretation of neural activities, mimicking the working functions of the brain and biological nervous systems [5]. Designing interactive artificial neuron systems that integrate sensing, storage, and processing capabilities has become a key focus in this research area. These interactive artificial neuron devices and systems are considered highly important in endowing BMIs with neuromorphic sensing and interactive characteristics, enabling them to solve more complex problems. Current advancements in artificial neurons primarily rely on electrophysiological signals, such as microelectrode arrays (MEA), to interpret and transmit neurological information by recording action potentials of individual cells and local field potentials [6]. For example, Professor Michael Brecht and his research team reported a single neuron stimulation method to precisely achieve evoked spike pattern control and measurement [7]. Such methods could make contributions to insights into the neuronal working principles and “decoding” of cortical spikes [8].

However, performing communication with neurons using the natural language—neurotransmitters—and developing chemically based devices are still major challenges ahead [9]. Neurotransmitters are endogenous chemicals that mediate chemical synaptic transmission, enabling brain function and shaping our everyday life and activity. Imbalance of neurotransmitters would cause multiple neurological disorders, such as Parkinson’s disease, major depressive disorder, and epilepsy [10]. Thus, a closed-loop chemical-to-electrical-to-chemical signal transduction system will provide us an alternative method for better communication with biological neural networks and modulation of their activities.

In contrast to conventional computer processors, artificial neurons have the ability to emulate the functions and operational patterns of neuronal activity in the human brain, enabling them to tackle more complex tasks. This field of research is interdisciplinary, involving material science, neuromorphic engineering, electronics/iontronics, electrochemistry, and neuroscience [5,8,11]. However, despite significant progress, these neuromorphic devices are still relatively new and face numerous challenges when it comes to establishing direct communication with biological neural networks. One of the key challenges in the development of artificial neurons is finding a way to achieve both bio-integrity and the ability to replicate living neural functions simultaneously. Addressing the requirements for materials, fabrication methods, and influential applications is of utmost importance. In this paper, we will outline these requirements for artificial neurons and provide an overview of the recent state-of-the-art advancements, along with their various applications.

We have organized this article into several sections, each addressing different aspects and requirements of artificial neurons. We start by discussing the necessary prerequisites and materials for these devices, followed by an exploration of their novel designs and potential uses. We then present a comprehensive conclusion summarizing the findings and advancements in the field. Additionally, we provide an outlook on future possibilities and the potential impact of these developments. Throughout the review, we emphasize the importance of supporting materials and unique fabrication methods that contribute to the success of these devices. The progress made in the development of such devices and systems has paved the way for the emerging flexible bioelectronic technology, which has the potential to expand the scope of current technologies in human–machine interfaces (HMI).

## 2. Materials and Fabrication Technology

As an implantable bio-integrated devices, the material selection and fabrication process of artificial neurons need to be chosen carefully in order to reach the high standard for long-term working inside human body. In this Section, we are going to discuss the material requirements and suitable fabrication technologies for artificial neuron devices and systems.

### 2.1. Material Requirements for Artificial Neurons

**Figure 1:**
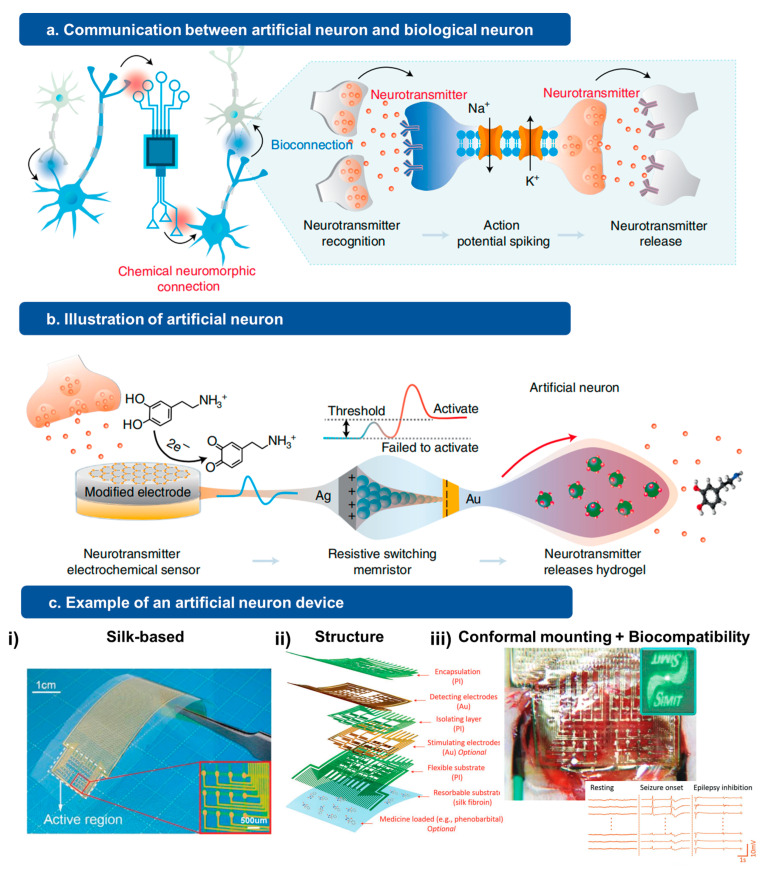
Schematic illustration of the artificial neuron key components. (**a**) Schematic of structure and working mechanism of biohybrid neurons, showing the interface between biological and artificial neurons. Such design could achieve neuromorphic communication and form a closed loop system. (**b**) Illustration demonstrating the detailed design of the artificial neuron and showing different components, including an electrochemical sensor, a signal processing unit, and a hydrogel-based molecular releasing part. Such design enables the device to detect and release neurotransmitters. Reprinted with permission from Ref. [12] Copyright 2022 Springer Nature. (**c**) Conformal brain electrodes. (i) Image of the brain electrode fabricated on silk film and the Au electrical probe array. (ii) Schematic of device configuration, showing the detailed structure of each layer in the device. (iii) Image of the device during the animal experiment when the brain electrode is implanted in the rat brain. The insect image shows the electrocorticography (ECoG) signals of different statuses, such as resting, epileptic seizure onset, as well as epilepsy inhibition statuses. The device also includes a silk-based diffractive optical elements (DOEs), as shown in the green inset picture. This optical component aims to achieve real-time monitor-like drug release processes and conformal mounting status. Reprinted with permission from Ref. [13] Copyright 2019 John Wiley and Sons.

The design and fabrication of artificial neurons draw inspiration from biological synapses and neural networks. To create a functional artificial neuron device, various components are required, including a data acquisition system that utilizes multi-modal bionic receptors for sensing, an artificial synapse for data processing and analysis, and an output unit for controlling biological neural networks. For illustration purposes, Figure 1 provides a visual representation of a basic artificial neuron structure.

To ensure the safe and effective long-term interaction with the human body, particularly with brain tissues, the materials used in artificial neurons need to have low mechanical mismatch and robust biocompatibility to minimize potential damage such as inflammation, infection, tissue intrusion, and allergic reactions [14]. Additionally, the multi-modal sensing capabilities of artificial neuron devices require materials that can be modified in various ways to capture multiple functions and achieve high sensitivity for accurate physiological monitoring [15,16]. Currently, there are several intrinsically biocompatible soft materials available that can serve as stretchable sensing components to record or respond to external stimuli. These materials include natural materials [17,18,19,20], polymers [21,22,23], hydrogels [24,25,26,27], and nanocomposite-based elastomers [28,29,30,31,32]. These materials can act as the fundamental building blocks of bionic receptors. In the following sections, we discuss several examples of these materials and highlight their key features that make them suitable for artificial neuron functions and applications.

In recent decades, there has been a growing need to develop materials that possess both robust bio-compatibility and flexibility for use in implantable bio-integrated artificial neurons, driven by advancements in technology and the desire to interface with diverse biological environments within the brain. As an example, silk materials have emerged as a versatile option for fabricating bio-integrated electronics, with numerous applications in tissue engineering and implantable medical devices [33,34,35,36]. Recent research has shown that silk-based materials exhibit strong adhesion to brain tissues, thanks to their unique mechanical properties and the ability to undergo chemical modifications [37,38]. Brain electrodes and devices incorporating silk-based materials have demonstrated excellent bio-compatibility and precise sensing capabilities.

Despite the promising qualities of silk protein and other natural materials, their usage is still in the early stages and presents several technical and commercial challenges that need to be addressed. These challenges include difficulties in reliable raw material processing and storage at a large scale, resulting in limited applicability [39,40]. Consequently, researchers frequently turn to synthetic materials that have already achieved large-scale production. One such example is the modified poly(3,4-ethylenedioxythiophene): polystyrene sulfonate (PEDOT:PSS) hydrogel, which has found extensive use in the development of bionic receptors and artificial synapses [41,42]. Organic neuromorphic devices that utilize PEDOT:PSS as the active material have successfully demonstrated synaptic functions such as short-term potentiation (STP) and global regulation of synaptic behavior. Furthermore, PEDOT:PSS can serve as a suitable surface for in vitro monitoring, and can also be utilized for the electrochemical detection of neurotransmitters through redox reactions. For instance, when dopamine is introduced into the electrolyte solution during the detection process, the conductance change is enhanced due to the oxidation of dopamine at the postsynaptic gate electrode.

### 2.2. Fabrication Methods for Artificial Neurons

In addition to the choice of high-quality materials, the selection of fabrication methods is a crucial step in the preparation of artificial neuron devices. Recent advancements in various fabrication techniques have greatly enhanced the capabilities of bio-integrated sensors. To effectively summarize the fabrication requirements for artificial neurons, it is important to differentiate the various components based on their specific functions. As illustrated in Figure 1, the structural components typically include the bionic receptors, the artificial synapse, and the output unit. Currently, in the case of implantable devices, soft materials still dominate as a major proportion of the fabrication process, as they provide enhanced flexibility and expanded functionalities compared to conventional sensing technologies. Furthermore, during the device fabrication process, careful consideration must be given to designing sensors that exhibit high sensitivity, fast response times, good stability, and tunable synaptic properties. Addressing these factors and incorporating them into the fabrication process will contribute to the overall performance and functionality of artificial neuron devices.

Bionic receptors, which are responsible for sensing chemical molecules in the surrounding environment, serve as a critical component of artificial neurons. Therefore, the development of new nanofabrication methods plays a vital role in enhancing the overall performance of these devices [39,43]. Extensive research efforts have been devoted to advancements in nanomaterial synthesis and device fabrication procedures, encompassing techniques such as inkjet printing, laser printing, optical microlithography, and hot-pressing modeling. For instance, a recent device fabrication method involving a chemically mediated artificial neuron consisting of three essential building blocks: a dopamine (DA) electrochemical sensor, a resistive switching memristor, and a heat-induced DA-releasing hydrogel [12]. A complete process flow was established, including the fabrication of a flexible substrate using a polyethylene terephthalate (PET) film, surface modification, and the incorporation of a polyvinyl alcohol (PVA)/silicon dioxide (SiO2)/DA hydrogel.

By employing these advanced nanofabrication techniques, researchers are able to enhance the performance and functionality of artificial neuron devices, ultimately leading to more precise and efficient chemical sensing capabilities.

Besides the bionic receptor, the memristor is also a crucial component in the construction of artificial neurons, responsible for information processing. Recent advancements in nanoscale material synthesis and corresponding nanofabrication techniques have enabled the development of more precise and advanced memristors. Typically, memristors utilize a metal–insulator–metal (MIM) structure, where the insulator layer is sandwiched between two metal electrodes.

To achieve multimodal sensing and computing capabilities, further improvements and developments in various aspects are necessary. These include techniques for decoupling multiple signals, designs for innovative device structures, and explanations for new sensing mechanisms. By continuously refining and enhancing these aspects, the performance and functionality of memristors can be improved, leading to more efficient and versatile artificial neuron systems.

## 3. Neuron Stimulation Technology

Apart from neuronal activity recording, neural stimulation is another fundamental component to construct an integral closed-loop feedback system for biological neural system regulation. Currently, the vast majority of treatments for neurological disorders are mainly based on stimulation technology, which involve oral administration of pharmaceuticals, electrical stimulation, and chemical stimulation.

Among them, the first two are widely used in the clinical treatment of neurological diseases, including epilepsy, Parkinson’s disease, depression, etc. However, the important factor is that they both can lead to various side effects, which can then affect physiological functions such as memory, learning, etc. In addition, drug resistance and dependence are also prevalent consequences that occur among neurological disease patients under certain prescription drug treatment. In comparison, local chemical stimulation, especially endogenous molecules such as neurotransmitters, is an ideal solution for these above-mentioned problems. Multiple devices have been successfully designed to achieve local chemical stimulation for neural network regulation. In this Section, we will demonstrate several methods that have been reported in recent research, including brain electrodes, microfluidic systems, a hydrogel-based releasing unit, and an organic electronic ion pump.

### 3.1. Brain Electrodes

Implanted brain electrodes are efficient methods to achieve electrical stimulation for neuronal activity modulation. Many groups have reported different brain electrode designs, which can stimulate the inactive biological neuronal network and modulate the brain functions. Some of them are now used in the clinical treatment for neurological disorder diseases like major depressive disorder, epilepsy, and Parkinson’s disease. However, such treatment sometimes can cause side effects, sometimes severely influencing patients’ daily activities. For example, deep brain stimulation and electroconvulsive therapy, which have been used as treatment methods for major depression, were associated with a risk of cognitive side effects, especially memory impairment. Although such side effects have been lowered due to technique improvement, they have not been successfully eliminated so far [44,45].

Furthermore, since the biological neurons’ activity is always based on chemical communications, electrical signals will cause a mismatch between living neurons and the artificial device and system, and find it hard to form an integral closed-loop feedback system. Thus, recent studies are more likely to focus on the chemical communication, especially for the endogenous molecules’ release methods for the artificial neurons, as shown below in the following sections.

### 3.2. Hydrogel-Based Releasing Unit

**Figure 2:**
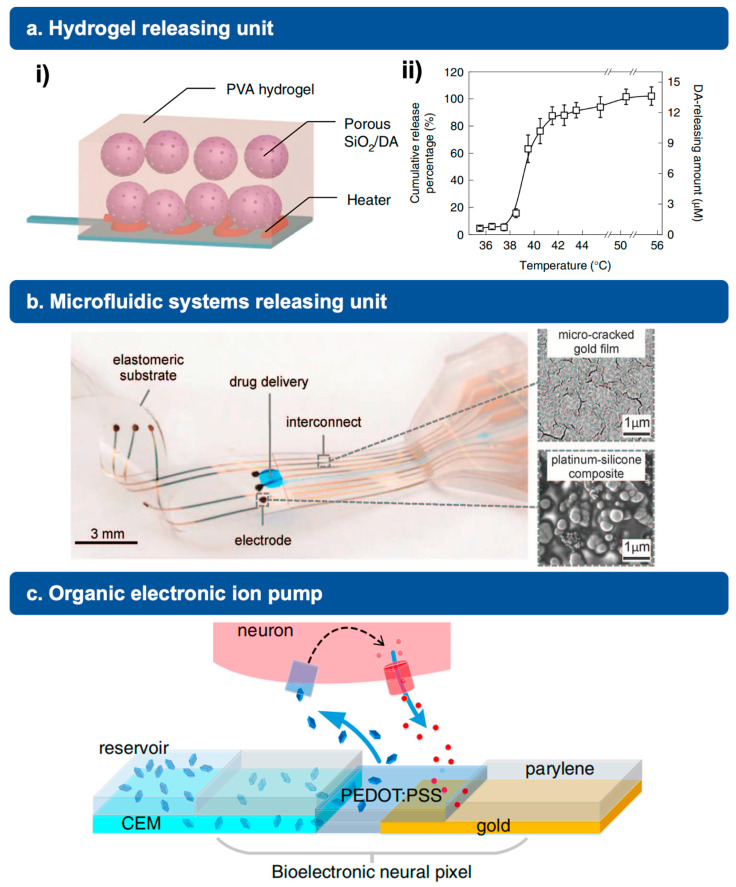
Examples of neuron stimulation technologies. (**a**) Hydrogel-releasing unit. (i) Schematic illustration of a temperature-sensitive dopamine release component, fabricated using PVA/SiO2/DA hydrogel. (ii) Results of dopamine release by the hydrogel-based unit. Reprinted with permission from Ref. [12] Copyright 2022 Springer Nature. (**b**) Optical image of an implantable drug delivery system fabricated on an elastomeric substrate and scanning electron micrographs of the platinum–silicone composite. Reprinted with permission from Ref. [46] Copyright 2015 The American Association for the Advancement of Science. (**c**) Illustration of an organic electronic ion pump based on PEDOT:PSS recording electrode and its working principle. The device is able to record the neurotransmitters and release molecules from the reservoir (left). Reprinted from Ref. [47].

Recently, several research groups focus on the study of chemically mediated hydrogel to achieve various drug molecules released for the therapeutic treatment of multiple diseases. This technique can also be used in artificial neural networks for biological neuron stimulation. For example, the research group of Xiaodong Chen has fabricated a heat-responsive hydrogel for the stimulation of dopamine (DA) release, forming a chemical communication loop between artificial neurons and biological neural networks, as shown in Figure 2a. In the experiment results part, they reported robust heat-responsive properties of this selected hydrogel, which can emulate controllable DA-releasing behavior in the axon vesicles [12]. Connected in series with a memristor, the deformation of the DA-encapsulated hydrogel can be triggered using a small potential (~0.5 V), allowing for the neurotransmitters to diffuse out of the PVA hydrogel and then fire the afferent nerve. Therefore, the system can achieve mediated DA release by adjusting the resistance value of the memristor.

Although this chemical-mediated hydrogel-based artificial neuron could enable bidirectional communication between artificial and biological neurons, there are still some features that need to be further improved, such as response time, power consumption, etc. In addition, this hydrogel-releasing design still has several unresolved problems that need to be fixed for practical applications. For instance, the heating process might cause damage to living cells and neurons when the temperature exceeds a certain value. Also, downscaling of the system, especially the hydrogel-releasing unit, and reducing the energy consumption need to be considered for long-term use. Lastly, as an implantable device system, the material’s and device’s stability need to be continually improved for integration with a complex neuron network.

### 3.3. Microfluidic Systems Releasing Unit

Another way to enable electrical-to-chemical signal transduction between artificial and biological neurons is microfluidic systems. Many studies have demonstrated the local release of endogenous molecules using microfluidics. Their basic design and structures are as shown in Figure 2b.

It is true that microfluidics is capable of delivering any soluble compound, providing large possibility for different molecules, drugs, and endogenous chemicals. Unfortunately, this system always requires the delivery of the target compound in a carrier fluid and will likely to induce convection, and might risk disrupting the fragile biochemical macrocondition. Although some groups have demonstrated a convection-free delivery system based on redox switching of the conducting polymer, the number of applicable molecules and compounds are still limited. In addition, complex and bulky set ups, such as pumps and valves, are usually inevitable, making the practical application restricted and inconvenient. Finally, the poor on–off rate ratio, release rate, and reaction speed are also disadvantages of this system which need improvements.

### 3.4. Organic Electronic Ion Pump

So far, a new technology, organic electronic ion pumps (OEIP), has recently started to be used in the design of bioelectronic neuron for the delivery of different endogenous neurotransmitters. Professor Daniel T. Simon and his research team reported a bioelectronic neuron pixel, which could achieve an inhibitory neurotransmitter, γ-aminobutyric acid (GABA) delivery, and stop epileptiform activity [42,47]. The OEIP can mimic the working principle of exocytotic release of neurotransmitters in biological neurons. In the design of OEIP, organic electrodes, normally made of conducting polymers, and permselective membrane are two key components, enabling precise neurotransmitters release without involving liquid flow, which is always necessary in microfluidic systems. In detail, as shown in Figure 2c, when a potential is applied between the two electrodes, one in reservoir and one in medium, an electric field will be established and the migration of neurotransmitters in the cation-conduction channel will be aroused. In the end, the process of record and release of GABA at the same site was demonstrated, and epileptiform activity of the downstream neurons can be suppressed successfully.

It is worth noting that, compared to the former mentioned types of neurotransmitter release methods, the structure of OEIP is quite compact and simple, avoiding any cumbersome and complex experimental set up, and can achieve recording and stimulating at the same site with high resolution. However, these systems are always limited in turn-on speed, since device dynamics are largely governed by the travel distance of delivered molecules. Thus, reducing the effective channel length and developing device with significantly faster turn-on are the areas that need more attention in the near future in order to improve the performance of OEIP for a better control of the release of neurotransmitters and neuronal activity modulation.

## 4. Synaptic Devices for Neural Signal Analysis

**Figure 3:**
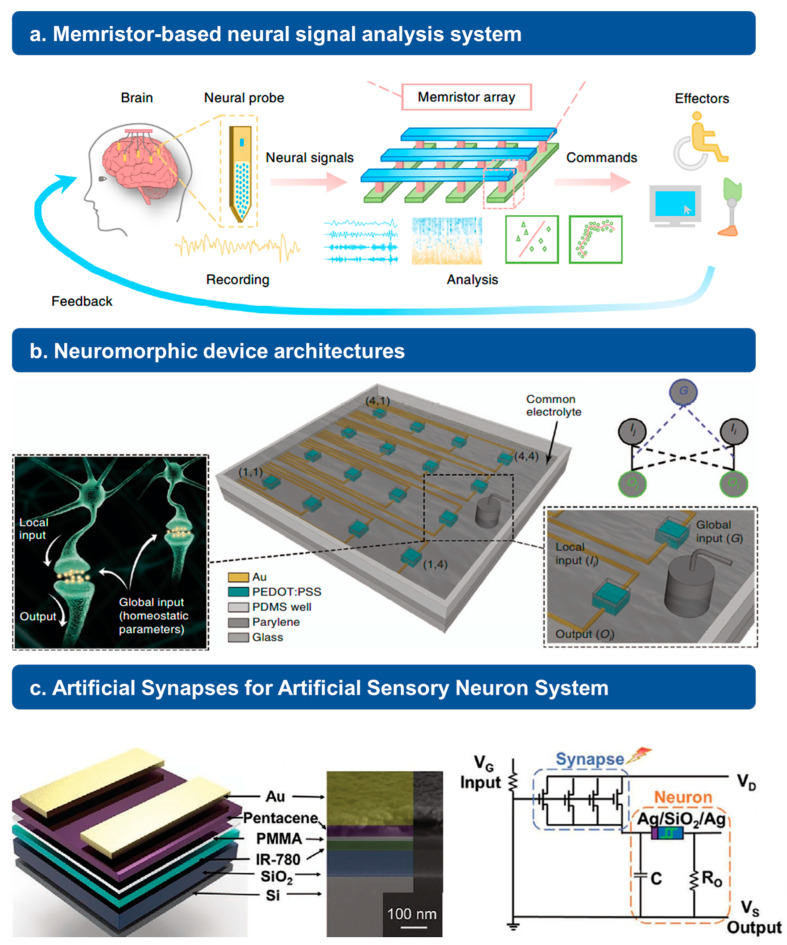
Synaptic devices for neural signal analysis. (**a**) Memristor-based neural signal analysis system for brain–machine interfaces (BMIs). Conceptual diagram shows the processes of recording neural activities and signal analyses based on a memristor array. Reprinted with permission from Ref. [48] Copyright 2020 Springer Nature. (**b**) Schematic of the configuration of the array of neuromorphic devices. Channel of each device is made of PEDOT: PSS. Reprinted with permission from Ref. [49] Copyright 2017 Springer Nature. (**c**) Schematic illustration and SEM image of synaptic transistor. Application of artificial sensory neuron system based on an array of artificial synaptic devices. Reprinted with permission from Ref. [50] Copyright 2021 John Wiley and Sons.

Apart from neural stimulation, analysis of signals after collection is also a critical part in order to form a closed-loop system used for human–machine interface applications. By mimicking the functions of biological synapses, properties like computing efficiency and speed can be greatly increased for a better application in bio-integrated intelligent systems. As shown in Figure 3, we provide an overview of synaptic devices used for neural signal analysis.

For example, Liu et al. has demonstrated successful seizure detection by using memristor-based brain-machine interfaces. The illustration in Figure 3a shows the concept of their system design and its detecting flow. In their report, the power efficiency of this synaptic-like memristor-based sensing system is 400 times than traditional CMOS circuits. It proves that in-memory computations on the memristor crossbar can help with the reduction in energy consumption [48]. Additionally, the research group of Prof. George G. Malliaras reported a neuromorphic device, which can behave like the homeoplasticity phenomena in the neural environment (Figure 3b). By using electrolyte gating to build intricate connections between devices, they also reveal the advantages of networks of neuromorphic devices with less hardwired connectivity [49]. In addition, Boyuan Mu et al. have established near-infrared artificial synapses to achieve biological synaptic functions, such as short-term and long-term plasticity and spike rate dependence based on electrical and optical properties’ modulation. The results showed in their work indicate that the development of artificial synapses could contribute to the areas of neural robots and neural computing [50].

## 5. Potential Applications

**Figure 4:**
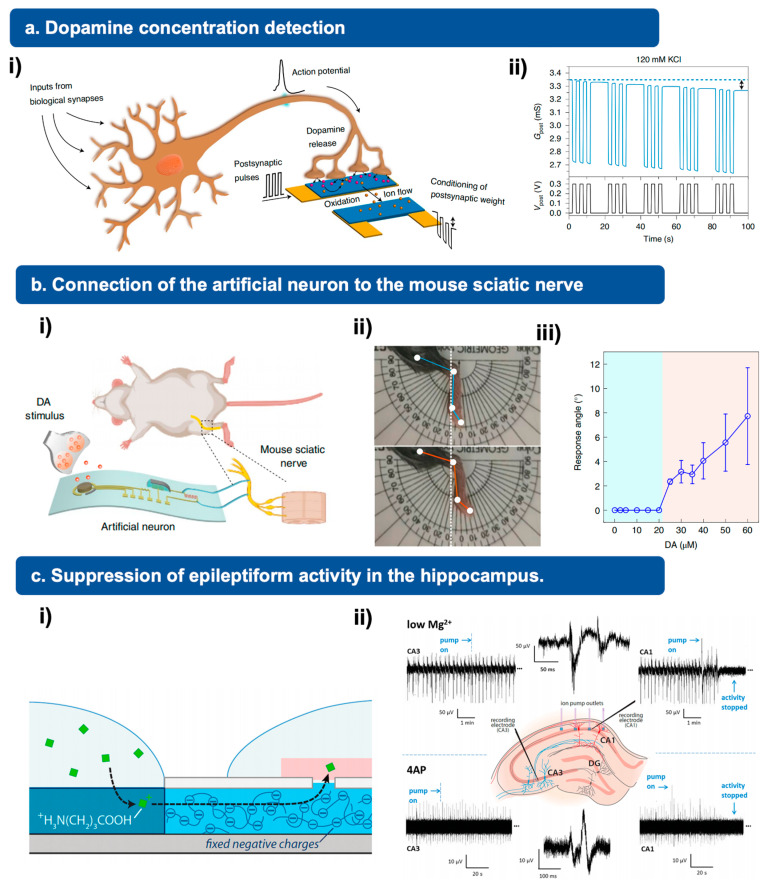
Different applications of artificial neuron. (**a**) (i) Schematic illustration of the potential interface between artificial neuron and biological neural network systems. (ii) Long-term potentiation of postsynaptic current of artificial neurons. Reprinted with permission from Ref. [51] Copyright 2020 Springer Nature. (**b**) (i) Application of artificial neurons for re-establishing the connection of a mouse sciatic nerve. (ii) When exposed to a dopamine stimulus, the artificial neuron can trigger the movement of the mouse leg and control the degree of such movement with different molecular concentrations. (iii) The response angle of the mouse leg under DA stimulus with different concentrations. Reprinted with permission from Ref. [12] Copyright 2022 Springer Nature. (**c**) (i) Device structures and working principles: positive GABA ions being delivered. (ii) Suppression of epileptiform activity in the hippocampus with OEIPs. Reprinted with permission from Ref. [52] Copyright 2015 John Wiley and Sons.

Artificial neurons have the ability to mimic the fundamental capabilities of biological neurons, such as sensing, processing, and regulating neuronal activities. Consequently, because of their ability for neuromodulation, they hold immense potential for various applications, including neuron rehabilitation, brain signal decoding, and human body control. Specifically, artificial neuron devices can be integrated with biological efferent nerves, or their engineered counterparts, to establish complete artificial or hybrid synaptic reflex arcs, as depicted in Figure 4.

As an illustration of the applications, the NTS (neurotransmitter-triggered synapse) can be seamlessly integrated with a motor/control unit, such as an insect leg, actuator, or pneumatic robot, to create a hybrid bioelectronic reflex arc that emulates the muscle activation process [53,54,55]. These advancements present significant potential in the fields of neurorobotics and neural prosthetics. In addition to interneurons, this system can also replicate the function of neurotransmitter-triggered motor neurons, enabling control over muscle contraction feedback. To exemplify this functionality, a dopamine (DA) stimulus is employed to initiate the movement of either a robotic hand or a mouse leg. Notably, the concentration of DA directly impacts the potential drop across the resistance (connected in series with the memristor), which in turn regulates the motion of both the robotic hand and the mouse leg. The experimental results demonstrate that in the absence of DA stimulus, neither the robotic hand nor the mouse leg can provide any feedback due to low input signals [12].

Furthermore, the team led by Christophe Bernard has made significant progress in utilizing three distinct models to induce epileptiform activity. They have successfully demonstrated that the administration of gamma-aminobutyric acid (GABA) leads to rapid and targeted suppression of this activity. This groundbreaking research suggests that these devices hold immense potential in terms of drug delivery within the brain, particularly for the administration of antiepileptic agents [52].

## 6. Conclusions and Outlook

To summarize, this review provides an introduction to the concept and fundamental structure of artificial neurons, offering an overview of recent advancements and emphasizing the key requirements and applications. We made a thorough summary of the recent development of artificial neurons, including the new materials, device architectures, fabrication techniques, and potential applications. To meet the criteria of artificial neurons, two crucial elements must be considered: the careful selection of appropriate materials, and the implementation of fabrication methods ensuring bio-integrity. Furthermore, the optimization of the paired materials’ preparation and fabrication processes is essential to seamlessly integrate diverse sensors and synaptic devices.

However, despite significant progress, there are still several challenges that need to be addressed in the near future. Firstly, the current detection limits are insufficient for accurately detecting sub-micromolar concentration changes in different neurotransmitters. Secondly, the bulky size of the sensing component hampers achieving precise spatial accuracy, thereby limiting the ability to achieve high-resolution molecule sensing. Thirdly, the level of device integration has not yet reached the stage of enabling simultaneous multichannel/multimodal sensing and processing. Fourthly, there is a lack of exploration in the areas of neuron adhesion, high flexibility, tissue compatibility, and their potential applications in understanding and treating depression mechanisms. These are all crucial aspects that require further research and improvement.

## Data Availability

The original contributions presented in the study are included in the article, further inquiries can be directed to the corresponding authors.
